# Organized thrombus is a frequent underlying feature in culprit lesion morphology in non-ST-elevation myocardial infarction. A study using optical coherence tomography and magnetic resonance imaging

**DOI:** 10.1007/s10554-023-03005-7

**Published:** 2023-12-21

**Authors:** Kathrine Ekström, Maria Radu Juul Jensen, Lene Holmvang, Francis Richard Joshi, Allan Zeeberg Iversen, Per Lav Madsen, Niels Thue Olsen, Frants Pedersen, Rikke Sørensen, Hans-Henrik Tilsted, Thomas Engstrøm, Jacob Lønborg

**Affiliations:** 1 Department of Cardiology, The Heart Centre, Rigshospitalet, Copenhagen University Hospital, Blegdamsvej 9, Copenhagen, DK-2100 Denmark; 2 Department of Cardiology, Herlev and Gentofte Hospital, University of Copenhagen, Herlev, Denmark

**Keywords:** Optical coherence tomography, Cardiac magnetic resonance, NSTEMI, Thrombus containing lesion, Plaque rupture

## Abstract

The concept that the culprit lesion in non-ST segment elevation myocardial infarction (NSTEMI) is caused by sudden plaque rupture with acute thrombus formation has recently been challenged. While angiography is an old gold-standard for culprit identification it merely visualizes the lumen contour. Optical coherence tomography (OCT) provides a detailed view of culprit features. Combined with myocardial edema on cardiac magnetic resonance (CMR), indicating acute ischemia and thus culprit location, we aimed to characterize culprit lesions using OCT. Patients with NSTEMI referred for angiography were prospectively enrolled. OCT was performed on angiographic stenoses ≥50% and on operator-suspected culprit lesions. Hierarchical OCT-culprit identifiers were defined in case of multiple unstable lesions, including OCT-defined thrombus age. An OCT-based definition of an organizing thrombus as corresponding to histological early healing stage was introduced. Lesions were classified as OCT-culprit or non-culprit, and characteristics compared. CMR was performed in a subset of patients. We included 65 patients with 97 lesions, of which 49 patients (75%) had 53 (54%) OCT-culprit lesions. The most common OCT-culprit identifiers were the presence of acute (66%) and organizing thrombus (19%). Plaque rupture was visible in 45% of OCT-culprit lesions. CMR performed in 38 patients revealed myocardial oedema in the corresponding territories of 67% of acute thrombi and 50% of organizing thrombi. A culprit lesion was identified by OCT in 75% patients with NSTEMI. Acute thrombus was the most frequent feature followed by organizing thrombus. Applying specific OCT-criteria to identify the culprit could prove valuable in ambiguous cases.

**Supplementary Information:**

The online version contains supplementary material available at 10.1007/s10554-023-03005-7.

## Introduction

The key pathophysiological trigger of acute coronary syndrome (ACS) is traditionally considered to be a sudden rupture of a thin-cap fibroatheroma (TCFA) with subsequent acute thrombus formation [1, 2] causing acute myocardial ischemia. The clinical equivalent is chest pain and ST-segment elevation myocardial infarction, (STEMI) or non-ST-segment elevation myocardial infarction, (NSTEMI), representing varying degrees of coronary artery (sub)occlusion [1, 2]. Recently, this classical paradigm was challenged by autopsy data showing that two-in-three thrombi in patients dying from sudden cardiac death showed signs of organizing thrombi during early healing [3].

Optical coherence tomography (OCT) is an intracoronary imaging technique with near-histological resolution that can resolve intracoronary pathologies related to the culprit lesions. Therefore, it is a valuable tool for in vivo assessment of coronary lesions. Pathophysiological features that have been associated with an angiographic culprit lesion on OCT include: acute thrombus, plaque rupture, plaque erosion, calcified nodule and dissection [4–13]. Notably, it is known that thrombi have different healing stages [1–3]. While OCT has been compared to histology with regard to healed/layered plaques [14], validation of the earlier healing stages is challenging and has not been investigated.

Currently, angiography is the preferred modality for culprit identification [15]. However, it carries an inherent limitation as it does not permit direct vessel wall evaluation and therefore, the use of angiography to identify the culprit is controversial. Although the angiographically identified culprit lesion in patients with NSTEMI has previously been evaluated by OCT [4–6, 8–13], the incidence and characteristics of OCT-based culprit lesions remain unclear.

Cardiac magnetic resonance (CMR) is a non-invasive method for detecting even brief periods of myocardial ischemia shown by oedema up to 7 days after the incident [16] signifying a culprit territory [16–18]. Moreover, CMR permits direct visualization of myocardial infarction [19]. Using a combination of OCT and CMR, it is possible to compare information about thrombus age by OCT with the age of infarction as determined by CMR.

To explore the classical NSTEMI paradigm in vivo, we therefore aimed to describe and characterize the features of a culprit lesion identified by OCT rather than angiography. We further sought to evaluate and describe the incidence and features of an OCT-based culprit lesions. Finally, we aimed to describe the morphology of the earlier thrombus healing stages as seen by OCT and validated by CMR.

## Materials and methods

We prospectively included patients with NSTEMI referred for PCI at Rigshospitalet, Copenhagen University Hospital, Denmark or Gentofte University Hospital, Denmark. To be eligible the operator must have identified a culprit lesion on angiography and OCT assessment of the angiographic culprit lesion was required to qualify for the study. A full list of all inclusion and exclusion criteria is provided in the Supplementary Methods section. Patients were enrolled based on contemporary guidelines for NSTEMI [15] and we aimed to include patents with the highest likelihood of a type 1 infarction [15, 20]. Accordingly, patients with high probability of having other causes of myocardial injury than acute myocardial infarction were excluded (e.g., sepsis, tachy-arrythmia, heart failure, severe anaemia or recent cardiac procedures) [15]. The trial is registered in clinicaltrials.gov (NCT03479593).

The study was approved by the Central Danish Ethics Committee (H-17,023,377) and performed in accordance with the Declaration of Helsinki. Written informed consent was obtained from all patients.

### OCT image acquisition and analysis

After angiography, OCT was performed on all visually estimated diameter stenoses ≥50%, and any operator-suspected culprit lesions irrespective of stenosis severity. A detailed description of angiography and OCT image acquisition and analysis is provided in Supplementary Methods. OCT frames were analysed at every 0.5 mm by two independent observers (KE and MJ) using a semi-automated software (QCU-CMS, version 4.69 (Medis, Leiden, NL). In cases of disagreement, consensus was reached. Each frame was classified according to plaque type as follows: normal vessel, fibroatheroma, fibrocalcific plaque, and fibrous plaque, according to international consensus standards [21].

### OCT culprit lesion: culprit identifiers

In the current paper, the designation of a lesion as OCT-culprit or OCT non-culprit refers to the diagnosis of “culprit” being made by OCT- rather than angiography, and subsequent characteristics described refer to this definition. To classify a lesion as an OCT-culprit lesion, we formulated a hierarchical OCT-based definition in which several key culprit identifiers could be present simultaneously, either within the same vessel or in different vessels in the same patient. The definition was based on a combination of information about lumen size and thrombus morphology. Accordingly, we defined a lesion as a conclusive OCT-culprit if one of following features was present (from highest to lowest considered severity): (1) acute thrombus occurring in a plaque rupture or in highest-grade stenotic lesions; (2) plaque rupture without thrombus; (3) organizing thrombus in lesions with higher degree of stenosis than criteria 4–6, in the absence of acute thrombus or plaque rupture; (4) erosion: acute thrombus in large lumens, in the absence of predilatation; (5) spontaneous dissection; (6) calcified nodule in absence of other culprit features. Consequently, in cases of simultaneously occurring acute thrombus in two separate lesions, both were considered culprits, and thus a double OCT-culprit was present. However, in case of simultaneously occurring plaque rupture and organising thrombus, respectively, in two separate lesions, the plaque rupture ranked higher than organising thrombus and was considered the only OCT-culprit.

**Fig. 1:**
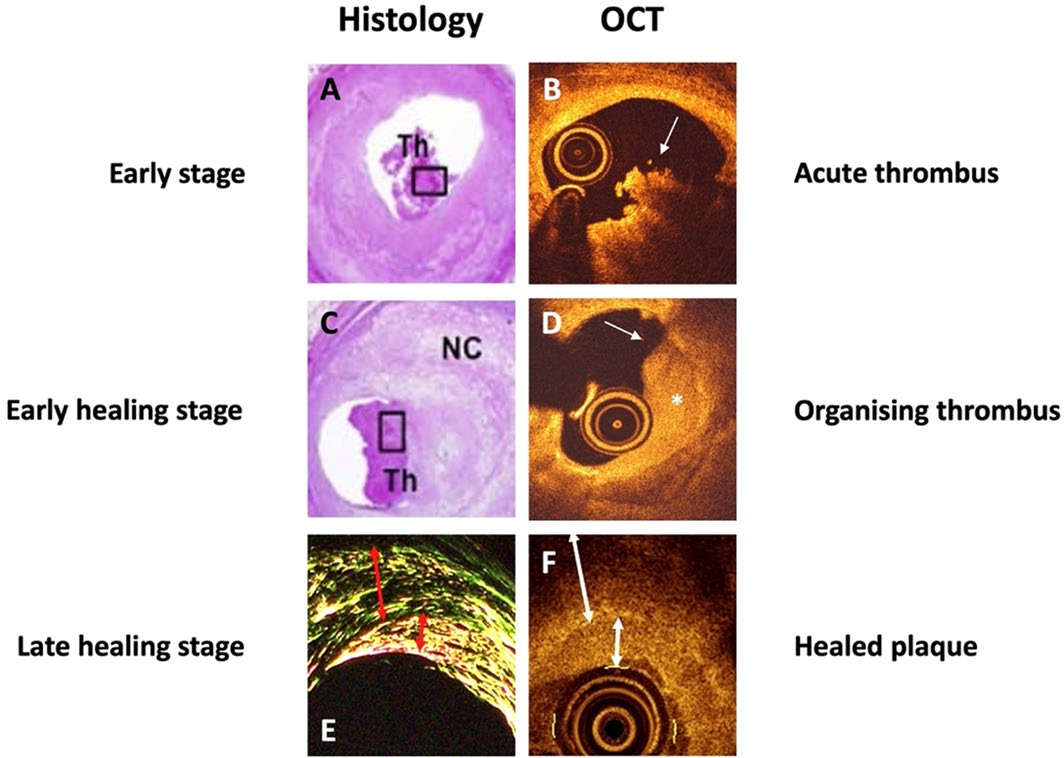
**Thrombus healing stages**. Histological thrombus healing stages: left panels. Reprinted with permission and adapted from Kramer et al. [3] and Shimokado et al. [14]. Histological early stage (**A**) and early healing stage thrombi (**C**) [3]. Late thrombus healing stage and healed/layered plaque visualised on histology (**E**) and OCT (**F**) [14].OCT-defined thrombus healing stages: right panels. **B** Acute thrombus: white thrombus with homogenous light intensity. A mass floating in the lumen or attached to the surface (arrow). Irregular, protruding with clear demarcation from the underlying tissue with irregular lateral delineation. **D** Organising thrombus: regular, rounded mass with a more concave surface and clear demarcation from underlying tissue (asterisk). The lateral delineation is more regular with a homogenous signal density (arrow) with layered appearance. **F** Healed/layered plaque: the surface is regular and rounded with a smooth lateral delineation (arrow **F**). The plaques are heterogenous; there is a clear demarcation to the underlying tissue. There is a signal-rich, bright light intensity compared with the underlying tissue (arrows **E**)

To establish OCT criteria for early thrombus healing stages, the inherent limitations of OCT and possible effects of medical pre-treatment had to be considered. Consequently, criteria for OCT-defined thrombus age were developed—incorporating key features of the pathological classifications that are visualisable by OCT in living patients; an extrapolation similar to what has been performed in other studies [4]. We noted remarkable morphological resemblances between published histological samples [3, 14] and our OCT-images (Fig. 1): in an OCT-context, we defined an *acute thrombus* as corresponding to a histological early-stage thrombus; an *organising thrombus* as corresponding to histological early healing stage; and finally, *late healing stage thrombus* as corresponding to histological healed/layered plaque [14]. The full definition is described in the Supplementary Methods section. Figure 1 shows the OCT-defined thrombus age as expected to correspond with previous published histological Sects. [3, 14]. Interobserver reproducibility regarding thrombus age was assessed and was substantial (kappa = 0.80 for acute and kappa = 0.75 for organising thrombus).

### CMR image acquisition and analysis

In a subset of patients, complementary CMR imaging was performed using a 1.5T scanner. The CMR protocol is elaborated in the Supplementary Methods. Briefly, we assessed myocardial edema using T2-weighted short tau inversion-recovery (STIR) images [19], and myocardial infarction using late-gadolinium enhancement (LGE) images [22]. Short-axis images covered the entire LV from the atrioventricular plane to the apex with contiguous 8-mm slices. CMR images were analyzed using dedicated software (CVI^42^ (Circle Cardiovascular Imaging Inc.), Calgary, Alberta, Canada) by an experienced reviewer (KE) and reviewed by a second, experienced independent reviewer (LNC) or by CMR team conference, all of whom were blinded to clinical, angiographic and OCT data.

A CMR culprit territory was defined as presence of edema by T2-STIR; ischemic-pattern LGE was not mandatory for a culprit diagnosis [16].

### Statistical analysis

To examine the difference between OCT culprit and non-culprit lesions, normally and non-normally distributed variables were compared accordingly using the Student’s T-test or Mann-Whitney test. Categorical variables were compared with a χ^2^-test or Fisher’s exact test as appropriate. Normal distribution was visually tested. We first examined the occurrence of OCT-culprit lesions. Additionally, we compared OCT-culprits with non-culprit lesions with respect to angiographic- and OCT characteristics including quantitative and qualitative measurements. Results were considered significant when below a two-sided P-value of 0.05. All analyses were performed with SPSS version 28 and GraphPad Prism version 9.

## Results

**Fig. 2:**
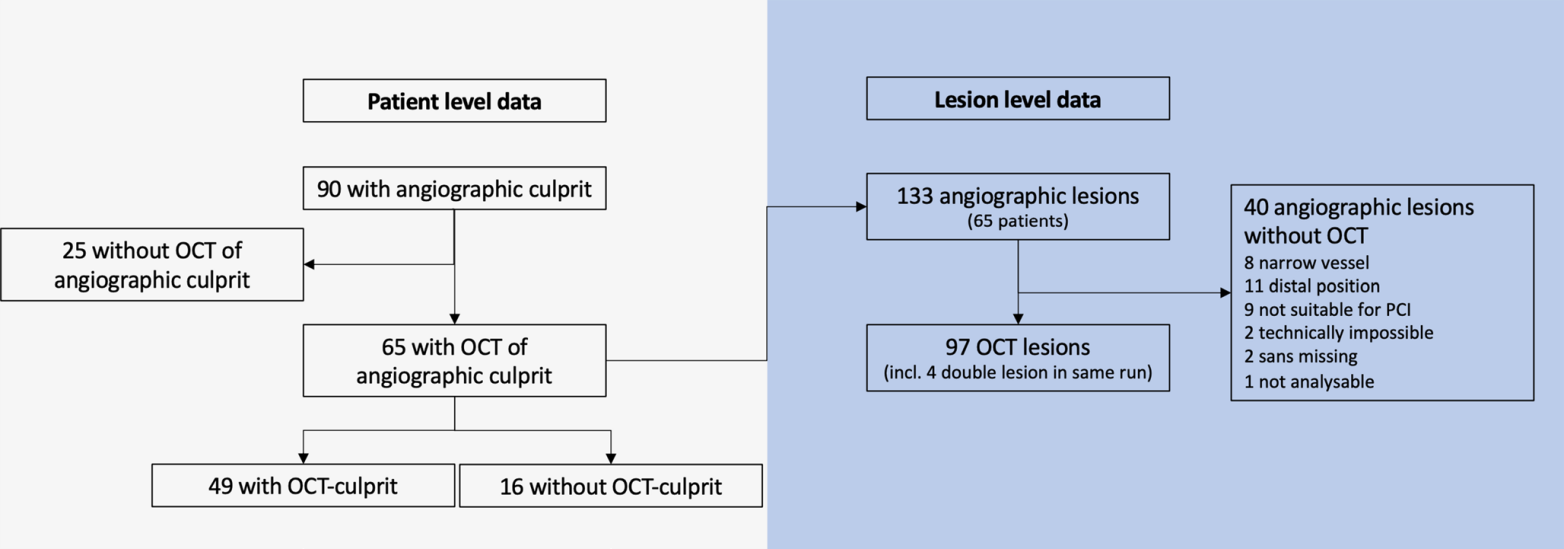
**OCT flowchart**. Patient level: of 90 patients with an angiographic culprit, OCT was performed in 65 patients who were included in this study. Of these, 49 had an OCT culprit. Lesion level: In 65 patients with OCT, 133 coronary artery lesions were identified on angiography. In 23 patients full OCT of all lesions was not possible in a total of 40 (30%) stenoses. Of the 93 angiographically lesions visualized with OCT, 4 double lesions were identified adding to a total of 97 lesions visualized with OCT. Full OCT was possible in 42 (65%) of the 65 patients

We included 65 patients with NSTEMI with 133 angiographic lesions, of which 93 were visualized using OCT. OCT was performed in the suspected angiographic culprit lesion in all patients. Overall, OCT was not assessable in 40 lesions primarily related to imaging inabilities such as distal lesion location and narrow vessel diameter (Fig. 2). Additionally, four angiographic lesions were found by OCT to be tandem lesions, leading to a total of 97 OCT lesions included in the study. Details regarding the inclusion flowchart and number of lesions imaged are shown in Fig. 2; Table 1 and Supplementary Results.

**Table 1:** Baseline characteristics in patients with one or more visible OCT culprit lesions and patients with no OCT culprit

	One/more OCT culprit visible, n = 49	No OCT culprit visible, n = 16
Age, years	63 ± 8	64 ± 11
Male sex, n (%)	40 (82)	10 (63)
Diabetes mellitus, n (%)	6 (12)	1 (6)
Hypertension, n (%)	28 (57)	9 (56)
Hyperlipidaemia, n (%)	22 (45)	7 (44)
Current/previous smoking, n (%)	30 (61)	10 (67)
Previous MI, n (%)	7 (14)	4 (25)
Previous PCI, n (%)	9 (18)	4 (25)
Family history of IHD, n (%)	20 (44)	6 (43)
Pre-admission angina pectoris (CCS ≥ 1)	36 (74)	12 (80)
**Admission medication**		
ASA, n (%)	14 (29)	4 (17)
Statin, n (%)	15 (31)	5 (31)
Clopidogrel or Ticagrelor, n (%)	2 (4)	1 (6)
(N)OAC, n (%)	2 (4)	0 (0)
**Laboratory data**		
Troponin or CKMB value above median for all values, n (%)	25 (51)	7 (44)
Significant rise and/or fall in troponin level, n (%)	48 (98)	15 (94)
**ECG findings**		
ST-segment depression	4 (8)	2 (13)
T-wave inversion	19 (39)	6 (38)
ST-segment depression + T-wave inversion	2 (4)	0 (0)
ST-segment elevation	2 (4)	0 (0)
No significant findings	14 (29)	7 (44)
Ischaemic ECG changes, n (%)	31 (63)	8 (50)
Symptom-to-wire time, days	4 (2–6)	3 (2-7.25)
Admission-to-wire time, days	3 (1.5-5)	3 (2-3.75)
Number of vessels imaged, n (%)		
1	26 (53)	10 (63)
2	21 (43)	5 (31)
3	2 (4)	1 (6)

Data are presented as n (%), mean (± SD) or median (IQR)

ASA, acetylsalicylic acid

Overall, patients were primarily male at a mean 63 years old. Ischaemic ECG changes were observed in 39 (60%) patients (Table 1). At patient level, OCT-features of a culprit lesion were found in 49 (75%) patients with 53 lesions; 4 (5%) patients had a double OCT-culprit lesion. Accordingly, an OCT-culprit was detected in 55% (53 of 97) of all evaluated angiographic lesions.

### Angiographic findings

Overall, 59 (91%) patients had at least one significant coronary stenosis (≥50%) in a major epicardial vessel, and the majority (n = 34, 52%) had multivessel disease. The visually estimated angiographic diameter stenosis was significantly worse in OCT culprit lesions (median 90% (IQR = 80;95%) vs. non-culprit lesions (80% (IQR = 50;90%) (p < 0.001) and there was a significantly higher proportion of lesions with compromised thrombolysis in myocardial infarction (TIMI) flow pre-PCI ($\le$2) in OCT culprit lesions compared with non-culprit lesion (n = 19, (36%) vs. 6, (14%), p = 0.02) There were no differences in the distribution of OCT- culprits in the three major coronary arteries compared with the non-culprit lesions (Table 2), nor were there any differences in the segmental location of OCT-culprit versus non-culprit lesions, but culprit lesions tended to be located more proximal, however not significant (Table 2 and online supplementary Fig. 1).

**Table 2:** OCT lesion level descriptive characteristics of OCT-culprit and non-culprit lesions

	OCT-culprit lesion, n = 53	OCT non-culprit lesion, n = 44	P-value
Lesion length, mm	16 ± 7	15 ± 8	0.37
Fibrous plaque, n (%)	24 (45)	22 (50)	0.64
Lipid plaque/fibroatheroma, n (%)	21 (40)	11 (25)	0.13
Lipid length, % lesion	45 (19–64)	34 (18–53)	0.24
TCFA, n (%)	3 (6)	0 (0)	0.25
Calcification, n (%)	7 (13)	6 (14)	1.0
Calcified length, % lesion	24 (14–48)	24 (12–50)	0.95
Presence of macrophages, n (%)	45 (85)	34 (77)	0.43
MLA, mm^2^	2.0 ± 2.0	2.0 ± 1.5	0.90
MLA lipid plaque, n (%)	31 (59)	16 (36)	0.03
MLA TCFA, n (%)	7 (13)	0 (0)	0.01
MLA calcification, n (%)	4 (8)	7 (16)	0.20
Minimal lumen diameter, mm	1.0 ± 0.5	1.2 ± 0.5	0.04
Diameter stenosis, %	74 ± 11	64 ± 11	< 0.001
**Angiographic data**			
Location of lesion within major arteries			
LAD, n (%)	28 (53)	18 (41)	0.31
Cx, n (%)	14 (26)	12 (27)	1.0
RCA, n (%)	11 (21)	14 (32)	0.25
Lesion located proximal, mid or distal			
Proximal, n (%)	22 (42)	10 (23)	0.06
Mid, n (%)	21 (40)	20 (46)	0.68
Distal, n (%)	10 (19)	14 (32)	0.16

Data are presented as n (%) or mean ±SD

Cx, left circumflex artery; LAD, left anterior descending artery; MLA, minimal luminal area; RCA, right coronary artery; TCFA, thin cap fibroatheroma

### OCT culprit identifiers and underlying plaque characteristics

**Fig. 3:**
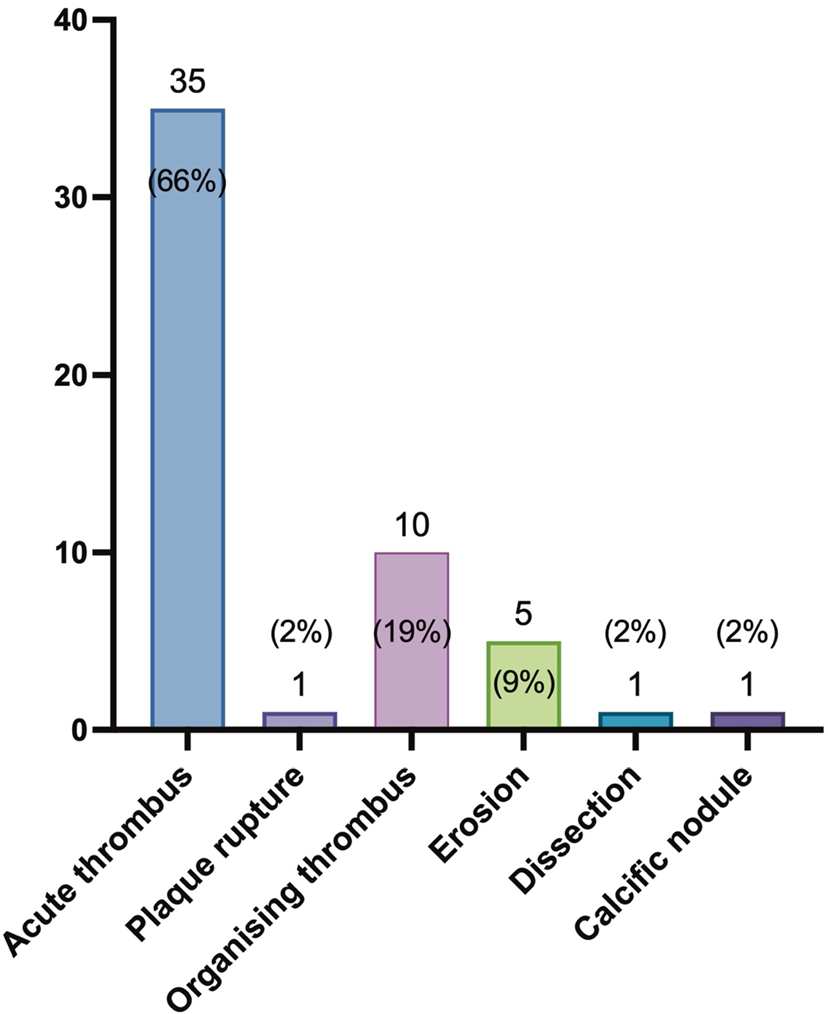
**Hierarchical culprit identifiers**. Occurrence of the main hierarchical culprit identifiers. Of note, several criteria could be present simultaneously. Numbers represents total number of cases and percentages refers to the number of culprit lesions

The frequency of the single features used to identify a culprit (Fig. 3), and the overall occurrence of all individual OCT-culprit features are reported. Accordingly, the most frequent OCT culprit identifiers according to the hierarchy (i.e., features thought to be responsible for calling the lesion an OCT-culprit) were the presence of acute (66%) or organizing thrombi (19%) (Fig. 3). Overall, OCT-culprits exhibited plaque ruptures in 45% of lesions, organizing thrombi and erosions in a total of 25% and 9% of lesions, respectively. Acute and organizing thrombi were present together in three OCT-culprit lesions (6%).

The minimal luminal diameter was significantly smaller (mean diameter 1.0 mm vs. 1.2 mm, p = 0.04) and the grade of luminal diameter stenosis on OCT was significantly higher (mean 74% vs. 64%, p < 0.001) in OCT-culprit compared with non-culprit lesions (Table 2). The lesion lengths were similar and there was no difference in the presence of macrophages between OCT-culprit and non-culprit lesions (Table 2). There was a significantly higher proportion of lipid plaques at the MLA, including TCFA, OCT-culprit compared with non-culprit lesions (Table 2).

### CMR versus OCT in thrombus age assessment

**Fig. 4:**
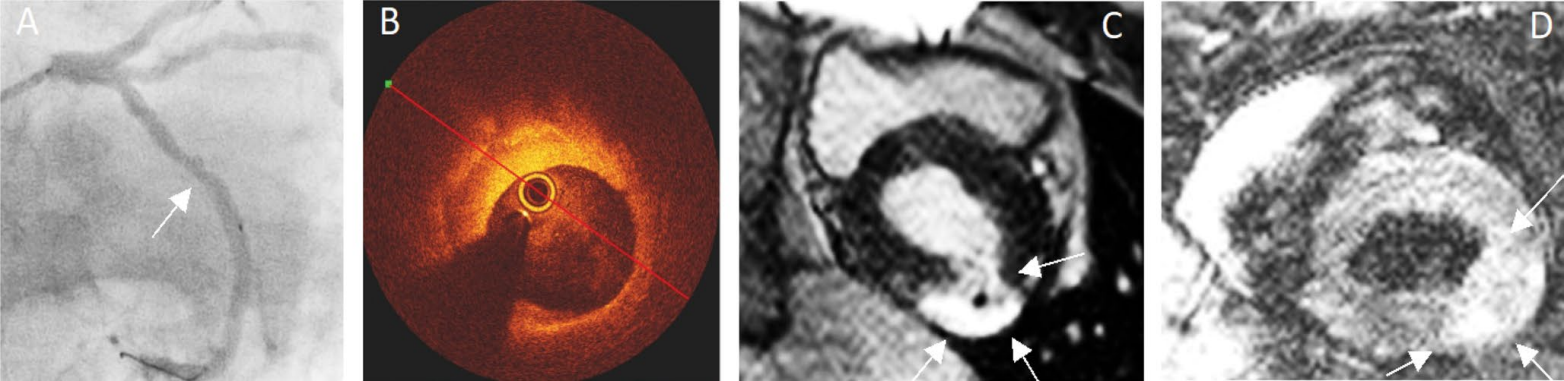
**Organizing thrombus as the culprit in NSTEMI**. Angiography of proximal LCX including OCT showing a 40% stenosis (**A**) and corresponding cross-sectional OCT (**B**) image shows an organizing thrombus due to a darker gradient towards the underlying luminal surface. Complementary CMR images (**C**; LGE image and **D**; T2-weighted image) of a single mid-ventricular short-axis slice: A large hyperenhanced area laterally (**C**, arrows) with a small black core indicating a large infarction with microvascular obstruction. The corresponding T2-weighted image (**D**) shows a hyperenhanced area with oedema laterally indicating acute infarction

Multimodality imaging with CMR and OCT were performed in 38 (58%) patients. In these, an acute thrombus on OCT was present in 18 (47%) patients, organizing thrombus as OCT-culprit identifier in 6 (16%) patients and healed plaques in 22 (58%) patients. Figure 4 shows an example of an organizing thrombus as the culprit in NSTEMI. Acute thrombi caused oedema in the corresponding myocardial territory in 67% of the patients and myocardial infarction in 50%. Likewise, in 50% of patients with organizing thrombi, oedema and acute myocardial infarction were present in the corresponding coronary territory (Table 3). Discordant findings were present only in two patients (5%): one acute thrombus with underlying chronic infarction and one healed plaque with underlying oedema and acute infarction (Table 3).

**Table 3:** Thrombus age and corresponding presence of oedema, acute and chronic infarction in the corresponding coronary artery territory

	Acute thrombus N = 18	Organising thrombus N = 6	Healed plaque N = 22
Oedema, n (%)	12 (67)	3 (50)	1 (5)
Acute infarction, n (%)	9 (50)	3 (50)	1 (5)
Chronic infarction, n (%)	1 (6)	0 (0)	1 (5)

Data are presented as n (%)

## Discussion

Identification of culprit lesions in NSTEMI is important but can be challenging. In the present study, we have challenged the conventional angiographic way of evaluating culprit lesions and rather assessed them using near-histological OCT. Accordingly, we defined a hierarchical approach assigning different importance to various OCT features and morphological findings. Our findings show that in addition to acute thrombus formation, organizing thrombus on OCT was a frequent identifying feature of a culprit lesion, supported by CMR.

There is currently no consensus regarding the definition of the culprit lesion, although it is much debated. Pathologists have defined the culprit as presence of thrombus or the tightest lesion in absence hereof [23], or more broadly––and highly pragmatic––as the lesion responsible for the current event [2]. Until now, in vivo studies have used readily accessible methods to identify the culprit: angiography, sometimes incorporating ECG and echocardiography [4–6, 8–13]. Therefore, definitions are far from uniform reflecting the need for a universal definition. Our OCT-based hierarchical definition based on the pathological characteristics attributable to an acute or very recent event. Moreover, OCT-defined acute thrombus alone has recently been used as a culprit identifier [24]. Notably, we found multiple culprit lesions in 5% of patients. Previous studies have found a wide range of multiple culprit lesions ranging from 2–35% [7, 11, 12, 25, 26]. Discrepancies are likely related to the definition of multiple culprits and populations examined. Multiple signs of instability may be present simultaneously––and because instability signs are also found in chronic coronary disease [27]––it is valuable to identify the actual culprit and differentiate it from bystander or precursor lesions, something that this study likely adds. Accordingly, and as recently proposed by OCT consensus [21] the use of OCT is pivotal to identify a culprit lesion(s) to ultimately guide treatment. In this context, both the treatment of the culprit and avoiding unnecessary treatment of non-ischemic non-culprit lesions are important.

Our findings corroborate that thrombus and plaque rupture are the main causes of NSTEMI [1, 2, 6, 7]. However, in contrast to previous in vivo studies that did not consider an organizing thrombus as a potential culprit identifier [21, 24], we found that as many as 25% of culprit lesions had thrombus with organized appearance. This novel OCT-definition using accepted, validated criteria [14, 21, 28] was based on extrapolation of histology-derived morphological features of thrombus healing stages, which is an approach also used by other research groups [4]. A validation of our findings with histology, if possible, is of course desirable. Morphologically, the organizing thrombus visualized by imaging bears very close resemblance to the histological early healing stage of a thrombus [3]. This is corroborated by two studies showing a high incidence of organizing thrombus in STEMI aspirates (> 50%) [29] and sudden cardiac death (two- thirds) [3]. Therefore, in some patients the *clinical* event could be a more dynamic pathophysiological process where the thrombus waxes and weans [30] before becoming symptomatic. This supports our definition of an organising thrombus as a culprit identifier in NSTEMI. Recently, numerous studies examining the healed plaque in ACS have emerged linking it with pan-vulnerability and stepwise plaque progression [12, 14, 31]. Compared with the healed plaque, an organising thrombus eventually becomes a part of the healed plaque entity and appear layered. The concept surfaced already in the 1990s by pathologists, [1] but has until now not been examined *in vivo.* Our findings confirm previous ex vivo studies, as we found organizing thrombi simultaneously with acute thrombi, suggesting a pan-vulnerable state.

Studies comparing CMR and OCT findings in the context of ACS are scarce. Results from a recent large study of women with myocardial infarction and non-obstructive coronary artery disease (MINOCA) [32] revealed that 75% of patients had a corresponding CMR and OCT-culprit. This included 42% of patients with CMR-detected myocardial infarction and a corresponding OCT culprit, which is in line with our findings. Moreover, we found that healed plaques infrequently had underlying chronic infarctions. This supports the theory that not all intravascular thromboses cause myocardial changes [12, 14, 31].

Particularly in ambiguous angiographic cases it is crucial to provide a correct diagnosis to avoid misdiagnosis and overtreatment. We identified an OCT-culprit lesion in three of four patients with a suspected angiographic culprit; this is comparable to the few studies reporting on identifiable culprit(s) using OCT in 69–85% of patients with NSTEMI. [4, 9] Consequently, it may not be possible to identify a culprit lesion in all patients with NSTEMI. First, due to imaging limitations; although, the majority of culprit lesions should be accessible for examination by OCT [33]. Other reasons include delay between symptom onset to examination, lesions not eligible for PCI, lysed thrombus, and coronary artery spasms not identifiable upon examination. Nevertheless, even considering these limitations it underlines the fact that OCT may be valuable for making the correct diagnosis in ambiguous cases, and OCT has already proven useful in establishing the diagnosis in patients with MINOCA [32].

### Limitations

Predilatation was allowed which may affect the OCT analysis. However, the morphological appearance of the typical tear, also seen in edge dissections [34], differs substantially from a spontaneous plaque rupture and we have accounted for the characteristics used in our definition. The lower prevalence of erosions (9% vs. 31–62% [4–6]) may be due to masking related to predilatation. Nevertheless, the risk of overlooking a culprit is very small since presence of thrombus would lead to a culprit diagnosis.

Furthermore, rather than performing systematic 3-vessel OCT, we aimed for a balance of invasively examining lesions with a higher probability of identifying a pathological culprit feature: (1) operator-suspected angiographic culprit lesions, and (2) any visually estimated significant lesion. The rationale behind was firstly pragmatic, and we aimed to examine lesions that may need revascularisation. Additionally, safety was considered, i.e., performing an invasive imaging procedure in vessels without angiographic lesions. Consequently, pathophysiological (OCT) culprit features occurring in non-significant stenoses may have been overlooked as these were not examined. However, our results are in line with pathology and in vivo findings, where culprit features have been identified in up to 85% [1, 4, 9] of patients with acute coronary syndrome or sudden cardiac death. Discrepancies may reflect the heterogeneity of NSTEMI, the populations examined, the diagnostic tools used, and finally, that patients with type 2 infarction could have been included. Furthermore, OCT was not possible in 30% of lesions primarily due to distal location and narrow vessels, introducing the risk of selection bias. However, we consider it less likely that a substantial number of culprit lesions were overlooked, as the majority are expected to be relatively proximal and thus should be reachable by OCT [33].

In conclusion, our results provide a deeper insight into the pathophysiology of NSTEMI, which should be considered a dynamic process. Using OCT, the most frequent features of a culprit lesion in NSTEMI were the presence of acute and organising thrombus, supported by CMR. Applying hierarchical OCT criteria, a culprit lesion was identified in three in four patients. In NSTEMI, an OCT-based identification of a culprit may assist angiography in ambiguous cases.

### Electronic supplementary material

Below is the link to the electronic supplementary material.

Supplementary Material 1
