# Supplementary material for: Organized thrombus is a frequent underlying feature in culprit lesion morphology in non-ST-elevation myocardial infarction. A study using optical coherence tomography and magnetic resonance imaging
Source: Int J Cardiovasc Imaging. 2023 Dec 21;40(2):441–9. doi: 10.1007/s10554-023-03005-7 (PMC10884357; doi:10.1007/s10554-023-03005-7)
Supplement: Supplementary file 1 — Supplementary Material 1 [file 10554_2023_3005_MOESM1_ESM.docx]

# Supplementary materials

Supplementary Methods.

An NSTEMI diagnosis was based on relevant symptoms of acute myocardial infarction, or new ischaemic ECG changes without persistent ST-segment elevation, and significant increase and/or decrease of cardiac biomarkers (troponin I/T, hs-troponin I or CK-MB).^1 2^ Patients with high probability of having other causes of myocardial injury than acute myocardial infarction were excluded (e.g., sepsis, tachy-arrythmia, heart failure, severe anaemia or recent cardiac procedures).^1^ Patients were excluded if no stenosis or suspected culprit lesion on angiography were found, or if OCT could not be performed for any reason (chronically occluded vessel, distal location of the stenosis, LM stenosis, narrow vessels not eligible for PCI). Careful predilatation with an undersized balloon was allowed if the OCT catheter could not be advanced and images otherwise not obtained. The operator was not blinded to the OCT scans which could be used for subsequent clinical decision making. All patients were treated according to contemporary guidelines.

*Angiography*

PCI was performed in accordance with current consensus standards^3^. The operator identified the culprit lesion on angiography based on one or more of the following criteria: intraluminal filling defect consistent with thrombus, plaque ulceration (haziness), reduced flow, recently occluded vessel, tightest lesion in the absence of any other criteria, or a clinical assessment based on available information (ECG and echocardiography).

*OCT*

Image acquisition was performed with the ILUMIEN or OPTIS systems with the Dragonfly Duo or Dragonfly Optis catheters (Abbott Vascular, Santa Clara, CA, USA) over a pullback length of 54-75 mm with an automated pullback speed of 10, 20 or 25 mm/sec at a frame rate of 100 frames/sec, with continuous flushing in the vessel by injection of contrast medium. Intracoronary nitroglycerin was administered prior to performing OCT.

OCT pullbacks were analysed by two independent observers: One observer (KE) did the primary analysis, which was then validated by a second and independent experienced observer (MJ) using dedicated software, QCU-CMS, version 4.69 (Medis, Leiden, NL). In case of disagreement, consensus was reached. Detailed analysis was performed every 0.5mm in the region of interest (ROI), defined as the minimal luminal area (MLA) in the relevant angiographic lesion ±5mm proximal and distal. If the lesion extended beyond, then analyses were continued until the end of the significant lesion. Matching between OCT and angiography was performed either using the proprietary co-registration feature––when available––or side branches and landmarks as reference.

Plaque types were analysed according to consensus standards^4^ and classified as follows: fibroatheroma (FA), fibrocalcific plaque (FC) and fibrous plaque (FP). It was defined in a frame if it reached >90 degrees of the vessel circumference. In brief, FA was defined as diffusely bordered signal-poor regions after differential diagnoses had been ruled out (i.e., macrophages, tangential signal drop-out, calcifications). In case of a possible TCFA the cap thickness was assessed using the semi-automatic cap measurement tool,^5^ and defined as cap thickness <65μm; thick cap FA (ThCFA) ≥65μm. FC was defined as plaques containing sharply delineated signal-poor areas; FP as a signal-rich homogeneous intima thickness >600μm. At lesion- and frame level we recorded the following: the plaque type (according to degree of severity: TCFA>ThCFA>FC> FP), presence of macrophages, luminal integrity and presence of thrombus. The plaque type at MLA was also noted. As opposed to analyses of stent thrombosis, with large thrombus mass, thrombus in the present study was generally smaller and analysis of underlying plaque type overall possible. Thus, typically a small thrombus mass or layered type thrombus were present enabling visualisation through the plaque because of altered light intensity. Frames with large thrombus burden >90 degrees of the vessel circumference were excluded. In case of previous stent implantation in the ROI, plaque analysis within the neointima was performed. Neoatherosclerosis was considered present when the neointimal thickness was >600μm, and plaque analysis was performed as for unstented vessels.

The luminal integrity was classified as being either intact, having a rupture with cavity (i.e., spontaneous), dissection (non-intact lumen without cavity in the absence of predilatation, i.e., spontaneous), tear (non-intact lumen without cavity in the presence of predilatation, i.e., iatrogenic) or erosion (irregular acute thrombosis in the absence of rupture or tear, i.e., spontaneous). Quantitative lumen area measurements were obtained with automated contour detection with manual correction if required. In case of multiple OCT-lesions within the same angiographic lesion, they were considered separate when the distance between ROIs was >5mm.

*OCT-based thrombus age definitions*

Histological “early stage” – OCT-defined “acute thrombus”: a convex mass attached to the luminal surface or floating within the lumen. The thrombus surface was irregular with clear demarcation to the underlying luminal surface (if visible). The optical signal intensity was either relatively homogenous with low attenuation and good demarcation (white thrombus), or with progressive shadowing (red thrombus)^4^. The transition to the luminal surface of the vessel laterally was sharp (Figure 1).

Histological “early healing stage” – OCT-defined “organizing”­­­­­­­­­ thrombus: a regular, rounded mass exhibiting a concave surface. The signal intensity was rich and homogenous, and a darker gradient towards the underlying luminal surface could be exhibited. There was a clear demarcation from the underlying tissue and the transition to the vessel wall laterally is more regular than above, but not smooth (Figure 1).

Histological “healed thrombus” – OCT-defined “healed/layered plaque”­­­­­­­­­: has previously been defined as a multi-layered structure with one or more heterogenous signal-rich layers of different signal intensity located close to the luminal surface with clear demarcation from the underlying tissue^6^. The surface is regular and clearly concave with a smooth lateral transition (Figure 1).

*CMR image acquisition and analysis*

Patients had CMR performed by using a 1.5T 8-channel body array coil scanner (Aera, Avanto or Espree scanner, Siemens, Erlangen, Germany). A short-axis plane was set up using scout images and electrocardiographic (ECG)-triggered breath-hold steady state-free precession images in 2-, 4-, and 3-chamber views. Short-axis images covered the entire LV from the atrioventricular plane to the apex with contiguous 8-mm slices. Myocardial oedema was assessed on short-axis images using T2-weighted short tau inversion-recovery (STIR) images^7^. Myocardial infarction was assessed as LGE images 10 minutes after injection of 0.1mmol/kg body weight of gadolinium-based contrast (Gadovist, Beyer Schering, Berlin, Germany) using an ECG-triggered inversion-recovery sequence^8^.

CMR images were analyzed using dedicated software (CVI^42^ (Circle Cardiovascular Imaging Inc.), Calgary, Alberta, Canada) by an experienced reviewer (KE) and reviewed by a second, experienced independent reviewer (LNC) or by CMR team conference, all of whom were blinded to clinical, angiographic and OCT data. Consensus was reached in case of disagreement. Presence of oedema and infarction was assessed as hyperenhanced myocardium T2 STIR images and LGE images respectively. Threshold for T2 STIR was for >2SD^9^ and >5SD for LGE^9^.

Supplementary results

Complete OCT evaluation of all angiographically visible lesions was possible in 42 patients. The remaining 23 patients were still included even though it was not possible to have OCT of all angiographically visible lesions for the reasons described in Figure 1, i.e., imaging ineligibility: distal lesion location and small vessel diameter. There were no differences in the number of lesions without visualisation by OCT between patients with or without a visible OCT-culprit (median 0 (IQR=0;1) vs. 0.5 (IQR=0;1.25); p=0.72) and the majority of patients had one- or two-vessel OCT performed (Table 1).

In the remaining 16 patients without any OCT culprit identifiers, complete OCT evaluation was not possible in 5 patients. Reasons include imaging ineligibility: distal lesion location, ineligibility for PCI, narrow vessels, or occlusions.

There were no differences between patients with acute or organising thrombus with regards to pre-admission antiplatelet or anticoagulation medication (41% vs. 25%, p=0.49), statin use (32% vs. 33%, p=0.95) or time from symptom onset to examination (4 vs. 4 days, p=0.32).

**Supplementary Figure 1. Occurrence of OCT-culprit and non-culprit lesions by coronary artery segment**

Occurrence of culprit lesions (A), non-culprit lesions (B) on OCT by coronary artery segment. A) Red circles indicate occurrence of culprit lesion in the segment. B) Green narrowing indicates occurrence of non-culprit lesion in the segment. Results are shown as number (% total lesions).

References

1. Collet JP, Thiele H, Barbato E, et al. 2020 ESC Guidelines for the management of acute coronary syndromes in patients presenting without persistent ST-segment elevation. *Eur Heart J* 2021;42(14):1289-367. doi: 10.1093/eurheartj/ehaa575 [published Online First: 2020/08/30]

2. Thygesen K, Alpert JS, Jaffe AS, et al. Fourth Universal Definition of Myocardial Infarction (2018). *J Am Coll Cardiol* 2018;72(18):2231-64. doi: 10.1016/j.jacc.2018.08.1038 [published Online First: 20180825]

3. Roffi M, Patrono C, Collet JP, et al. [2015 ESC Guidelines for the management of acute coronary syndromes in patients presenting without persistent ST-segment elevation. Task Force for the Management of Acute Coronary Syndromes in Patients Presenting without Persistent ST-Segment Elevation of the European Society of Cardiology (ESC)]. *G Ital Cardiol (Rome)* 2016;17(10):831-72. doi: 10.1714/2464.25804 [published Online First: 2016/11/22]

4. Johnson TW, Raber L, di Mario C, et al. Clinical use of intracoronary imaging. Part 2: acute coronary syndromes, ambiguous coronary angiography findings, and guiding interventional decision-making: an expert consensus document of the European Association of Percutaneous Cardiovascular Interventions. *Eur Heart J* 2019;40(31):2566-84. doi: 10.1093/eurheartj/ehz332 [published Online First: 2019/05/22]

5. Radu MD, Yamaji K, Garcia-Garcia HM, et al. Variability in the measurement of minimum fibrous cap thickness and reproducibility of fibroatheroma classification by optical coherence tomography using manual versus semi-automatic assessment. *EuroIntervention* 2016;12(8):e987-e97. doi: 10.4244/EIJV12I8A162 [published Online First: 2016/10/11]

6. Shimokado A, Matsuo Y, Kubo T, et al. In vivo optical coherence tomography imaging and histopathology of healed coronary plaques. *Atherosclerosis* 2018;275:35-42. doi: 10.1016/j.atherosclerosis.2018.05.025 [published Online First: 2018/06/03]

7. Abdel-Aty H, Zagrosek A, Schulz-Menger J, et al. Delayed enhancement and T2-weighted cardiovascular magnetic resonance imaging differentiate acute from chronic myocardial infarction. *Circulation* 2004;109(20):2411-6. doi: 10.1161/01.CIR.0000127428.10985.C6 [published Online First: 2004/05/05]

8. Friedrich MG, Abdel-Aty H, Taylor A, et al. The salvaged area at risk in reperfused acute myocardial infarction as visualized by cardiovascular magnetic resonance. *J Am Coll Cardiol* 2008;51(16):1581-7. doi: 10.1016/j.jacc.2008.01.019

9. Bondarenko O, Beek AM, Hofman MB, et al. Standardizing the definition of hyperenhancement in the quantitative assessment of infarct size and myocardial viability using delayed contrast-enhanced CMR. *J Cardiovasc Magn Reson* 2005;7(2):481-5. doi: 10.1081/jcmr-200053623 [published Online First: 2005/05/11]
